# Plasmid encoded *mrk* gene cluster promotes biofilm formation, environmental persistence, and invasiveness in *Salmonella* Thompson

**DOI:** 10.1016/j.bioflm.2026.100394

**Published:** 2026-08-26

**Authors:** Ke Liu, Liya Feng, Lu Ouyang, Xinpeng Li, Xiaohong Shi, Liang Chen, Mingju Hao

**Affiliations:** a Department of Clinical Laboratory Medicine, The First Affiliated Hospital of Shandong First Medical University & Shandong Provincial Qianfoshan Hospital, Clinical Research Center for Respiratory Disease, Jinan, China; b NHC Specialty Laboratory of Food Safety Risk Assessment and Standard Development (Food Microorganisms), Shandong Academy of Preventive Medicine, Shandong Center for Disease Control and Prevention, China; c School of Pharmacy and Pharmaceutical Sciences, University at Buffalo, Buffalo, NY, 14214, USA

**Keywords:** *Salmonella* Thompson, Biofilm formation, *mrkABCDF*, IncX1 plasmid, Virulence and dissemination

## Abstract

Biofilm formation is a fundamental survival strategy that enables bacterial persistence across diverse environments and hosts. While the *mrk* gene cluster encoding type 3 fimbriae is a well-established determinant of biofilm formation in *Klebsiella pneumoniae,* its presence and functional significance in *Salmonella enterica* remains poorly defined. In this study, we identified a plasmid-encoded *mrkABCDF* gene cluster carried on a highly conjugative IncX1 plasmid in a clinical *Salmonella* Thompson isolate. Using CRISPR/Cas9-mediated knockout of the *mrk-*containing *Tn*6011 transposon, plasmid curing, targeted gene deletion, and genetic complementation, we systematically dissected the contribution of *mrkABCDF* genes to biofilm development and associated phenotypes. Loss of the *mrk* gene cluster resulted in a profound reduction in biofilm biomass and a concomitant increase in bacterial motility. Type 3 fimbriae were detected exclusively on the surface of *mrk-*positive cells, confirming their structural role in surface attachment. The *mrk* operon was strongly expressed from an IS1-associated promoter, bypassing canonical MrkH-dependent regulation. Functionally, *mrk* expression enhanced bacterial tolerance to desiccation and oxidative stresses, and reduced susceptibility to macrophage phagocytosis. In vivo, *mrk-*positive strains exhibited enhanced gastrointestinal colonization and tissue invasion. Notably, carbapenems exhibited exceptional efficacy in inhibiting *mrk-*mediated biofilm formation, indicating their superior potential for treating biofilm-associated infections. Our findings demonstrate that plasmid-encoded *mrkABCDF* genes can act as key architectural and functional determinants of biofilm formation in *Salmonella enterica*. The horizontal dissemination of *mrk-*carrying IncX1 plasmids may promote the emergence of biofilm-adapted *Salmonella* lineages with enhanced environmental persistence and host colonization potential.

## 1 Introduction

Non-typhoidal *Salmonella* (NTS) is a major global health threat, responsible for the majority of gastroenteritis cases worldwide, with an estimated 93.8 million infections and approximately 155,000 deaths annually [1]. Transmission most often occurs through food contaminated by carriers or their feces, leading to widespread foodborne illness [2]. Beyond self-limiting gastroenteritis, invasive non-typhoidal *Salmonella* (iNTS) poses an even greater public health challenge, as it can cause severe, life-threatening systemic infections. According to the Global Burden of Disease (GBD) study, iNTS accounted for approximately 509,000 incident cases worldwide in 2021 [3]. These figures underscore the significance of iNTS as one of the most prevalent and hazardous gastrointestinal pathogens globally.

Biofilm formation plays a critical role in the pathogenesis of iNTS by enhancing bacterial persistence, protecting against host defenses and antibiotics, and facilitating chronic colonization of intestinal and hepatobiliary niches, which can predispose to systemic invasion [[4], [5], [6]]. Biofilms are structured microbial communities attached to surfaces and encased in an extracellular matrix composed of polysaccharides, extracellular DNA, and proteins. This mode of growth provides protection against environmental stressors, antibiotics, and host immune defenses, thereby enabling long-term colonization within the host [7,8].

Among the genetic determinants of biofilm formation, the *mrk* gene cluster is well characterized in *K. pneumoniae.* This operon (*mrkABCDF*) encodes type 3 fimbriae, which mediate adhesion to surfaces and drive biofilm development [8,9]. Type 3 fimbriae not only provide structural stability to biofilms but also enhance bacterial persistence by conferring protection against antibiotics and host immune defenses. Within the cluster, *mrkA* encodes the major structural subunit, *mrkD* and *mrkF* function as tip adhesins, *mrkB* and *mrkC* are involved in fimbrial assembly and regulatory signaling [10,11]. These genes play a pivotal role in colonization and establishment of persistent infections in *K. pneumoniae* [12].

In our preliminary work, we identified an NTS strain isolated from a diabetic patient with a foot ulcer [13]. This strain demonstrated strong biofilm-forming capacity in vitro. Despite multiple courses of antibiotic therapy, three toe amputations, and flap transplantation, the infection continued to progress, ultimately necessitating complete foot amputation due to extensive tissue necrosis. Genetic analysis revealed that the strain carried the *mrkABCDF* gene cluster within the *Tn*6011 transposon, located on an IncX1-type plasmid. These observations highlight the clinical relevance of *Salmonella* strains harboring the *mrk* gene cluster and suggest that plasmid-mediated acquisition of type 3 fimbriae may enhance pathogenic traits beyond those traditionally associated with this species.

However, the dissemination dynamics of IncX1 plasmids carrying *mrkABCDF* genes in *Salmonella enterica* and their precise contribution to biofilm formation, virulence, and host colonization remain poorly understood. To address this gap, the present study investigates the molecular mechanisms by which the IncX1 plasmid–encoded *mrk* cluster influences biofilm development, environmental adaptability, invasiveness, intestinal colonization, and antibiotic resistance in non-typhoidal *Salmonella enterica,* thereby providing new insights into the role of horizontal gene transfer in shaping pathogenic potential.

## 2 Materials and methods

### 2.1 Bacterial strains

The clinical isolate (JNQH950) examined in this study was obtained from the foot exudate of a 58-year-old patient with diabetic foot infection at a tertiary hospital in China. The patient presented with a severe skin infection that was refractory to multiple courses of antimicrobial therapy, and non-typhoidal *Salmonella spp*. strains were repeatedly identified by routine culture. Despite intensive treatment, disease progression necessitated the right foot amputation, after which the patient achieved full recovery. Phenotypic characterization of the isolate revealed a strong biofilm-forming capacity. Whole-genome analysis further demonstrated the presence of an IncX1-type plasmid carrying the *mrkABCDF* gene cluster (pJNQH950-3). Serotyping based on the White–Kauffmann scheme identified the strain as *Salmonella enterica* serovar Thompson [13].

### 2.2 Comparison of genomic context of mrkABCDF in IncX1 plasmid and *Klebsiella pneumoniae*

To define the genetic environment of the *Salmonella* Thompson *mrk* locus, the complete 40,394-bp IncX1 plasmid pJNQH950-3 was mapped and compared with the archetypal *Klebsiella pneumoniae* sequence cluster (KpSC) locus represented by strain MGH 78578. Amino acid homology within the *mrkABCDF* operons was assessed using BLAST. Promoter regions upstream of *mrkA* were aligned, and sequence variations were analyzed.

### 2.3 mrk gene cluster promoter activity assay

To evaluate potential differences in promoter activity between the upstream regions of the *Salmonella* IncX1-borne and KpSC *mrkA* loci, promoter fragments were amplified using primer pairs BamH-373mrk-R/*Hin*dIII-373mrk-F and *Hin*dIII-950mrk-F/BamH-950mrk-R, respectively. The fragments were cloned upstream of sfGFP in the pPROBE-GT vector via *Hin*dIII and *Bam*HI restriction sites [14], followed by transformation into *E. coli* DH5α competent cells. Positive clones were confirmed by PCR and Sanger sequencing. For each replicate, green fluorescence intensity was normalized to the corresponding OD_600_ value to account for differences in cell density. The normalized activity of each promoter construct was then expressed relative to the mean normalized activity of the empty-vector control, which was set to 1.

### 2.4 CRISPR-Cas9-mediated deletion of the mrk-carrying Tn6011 transposon and IncX1 plasmid

Deletion mutants were generated using a CRISPR–Cas9–based plasmid-curing system derived from the previously described pCasCure vector (Table 1) [15,16]. A targeting plasmid, pCasCure-N20_*mrkA*_, was constructed to disrupt *mrkA*. Single-guide RNAs (sgRNAs) were designed using Geneious Prime software [17]. Using pCasCure as the template, the sgRNA cassette was amplified by primer pairs SpeI_mrkA/*Xba*I-R with the introduction of *Spe*I and *Xba*I restriction sites. The PCR product was digested with *Spe*I and *Xba*I and ligated into the similarly digested pCasCure vector to generate pCasCure-N20_*mrkA*_. Correct insertion of the N20 targeting sequence was confirmed by PCR (mrkA_N20/N20_188R) and Sanger sequencing (Table S1).

**Table 1:** Strains or plasmids used in this study.

Strains or plasmids	Relevant phenotype or genotype
**Strains**	
JNQH950	Clinical strain of nontyphoid *Salmonella* serotype Thompson
JNQH1093	JNQH950△IncX1
JNQH1100	JNQH950△*Tn*6011
JNQH1304	JNQH950 IncX1:*aac(3′)-IV*
JNQH1402	JNQH950*△mrkCD::aac(3′)-IV*
JNQH1115	JNQH1093:pEASY-*mrkABCDF*
*E.coli* WM3064	Δ*dapA*, *Sm*^*r*^*,* RP4 mobilization machinery inserted in chromosome. DAP auxotrophy
**Plasmids**	
pPROBE-GT	*rrnB T1 terminator, Gen*^*r*^*, sfGFP,* MCS
pCasCure	*aac(3′)-IV, ColE1, araC, Cas9, sgRNA, J23119*
pCasCure-N20_*mrkA*_	pCasCure with a N20 spacer targeting *mrkA*
pEASY-T&B cloning vector	*pUC* origin, *Kana*^*r*^*, Amp*^*r*^*, LacZα*
pLZ002-sGFP	*pUC origin, Kana*^*r*^*, Apm*^*r*^*, sfGFP*
pSZU941	*Apm*^*r*^ flanked by *Tn*7 mobile element, *oriT*
pSZU941-GFP	*sfGFP*, *Apm*^*r*^ flanked by *Tn*7 mobile element, *oriT*
EU215432	*TnsABCD, R6K ori, oriT*, Amp^r^
pGGTOX	*R6K ori, RP4, rhaS, Apm*^*r*^*, pRha, sfGFP, mqsR,* type II MCS
pLuci	*Amp*^*r*^*, Luciferase*

The verified plasmid was introduced into the recipient strain by electroporation. Single colonies were selected and cultured overnight in LB broth supplemented with 0.1% (w/v) arabinose to induce *Cas9* expression. Cultures were diluted and plated on LB agar containing apramycin. Deletion of the *mrkA* or the complete IncX1 plasmid was confirmed by PCR amplification of the *mrkA* and the IncX1 replicon. Subsequently, sucrose counterselection was applied to activate the *sacB* gene to eliminate the pCasCure plasmid. The strain cured of the entire IncX1 plasmid was designated JNQH1093, whereas the strain with selective deletion of the *mrkABCDF-*carrying *Tn*6011 transposon was designated JNQH1100 (Table 1).

### 2.5 Pulsed-Field Gel Electrophoresis (PFGE)

S1-PFGE was used to confirm the plasmid curing effect of IncX1 plasmid from JNQH950. PFGE of S1-digested genomic DNA samples were performed with a CHEF MAPPER XA apparatus (Bio-Rad, USA), as described in our previous research [18].

### 2.6 Complementation of the mrk gene cluster

The complete *mrkABCDF* gene cluster was amplified by PCR using primer pairs mrk-locL/mrk-locR. The purified PCR product (60 ng) was ligated into the pEASY-T&B cloning vector (9 ng) using the pEASY-T&B Cloning Kit and incubated at 37 °C for 20 min. The ligation mixture (5 μL) was transformed into *E*. *coli* DH5α competent cells, which were plated on LB agar containing kanamycin, IPTG, and X-gal and incubated overnight. White colonies were selected by blue–white screening [19]. Positive clones were verified by colony PCR, and the recombinant plasmid was purified and introduced into electrocompetent JNQH1093 cells by electroporation. The resulting complemented strain was confirmed by PCR amplification of the *mrk* gene cluster (Table S1).

### 2.7 Biofilm formation assay

Biofilm production was assessed using crystal violet staining as described previously with minor modifications [20]. Briefly, overnight bacterial cultures were diluted 1:1000 in LB medium, and 100 μL aliquots were dispensed into vinyl U-bottom 96-well plates. After incubation at 37 °C for 24 h, wells were washed four times with distilled water to remove non-adherent cells. Attached bacteria were stained with 125 μL of 0.1% crystal violet for 10 min, followed by six washes with distilled water. The bound dye was solubilized with 150 μL of 30% glacial acetic acid for 10 min at room temperature, and absorbance was recorded at 590 nm using a Thermo Scientific microplate reader. Each condition was tested in triplicate.

### 2.8 Bacterial swimming assay

The swimming motility of strains JNQH950 (WT), JNQH1093 (ΔIncX1), JNQH1100 (Δ*Tn*6011) and JNQH1115 (ΔIncX1-pEASY-mrk), was assessed using a soft-agar motility assay. Each strain was grown overnight in LB broth at 37 °C with shaking. The following day, cultures were adjusted to a turbidity equivalent to a 0.5 McFarland standard. Subsequently, 2 μL of each standardized culture was inoculated onto the center of LB plates containing 0.3% agar. Inoculated plates were incubated at 37 °C for 6 h. Following incubation, the diameters of the motility halos were measured to quantify swimming ability. The assay was tested in triplicate.

### 2.9 Insertion of resistance marker into mrk-IncX1 plasmid for horizontal transfer analysis

**Fig. 1:**
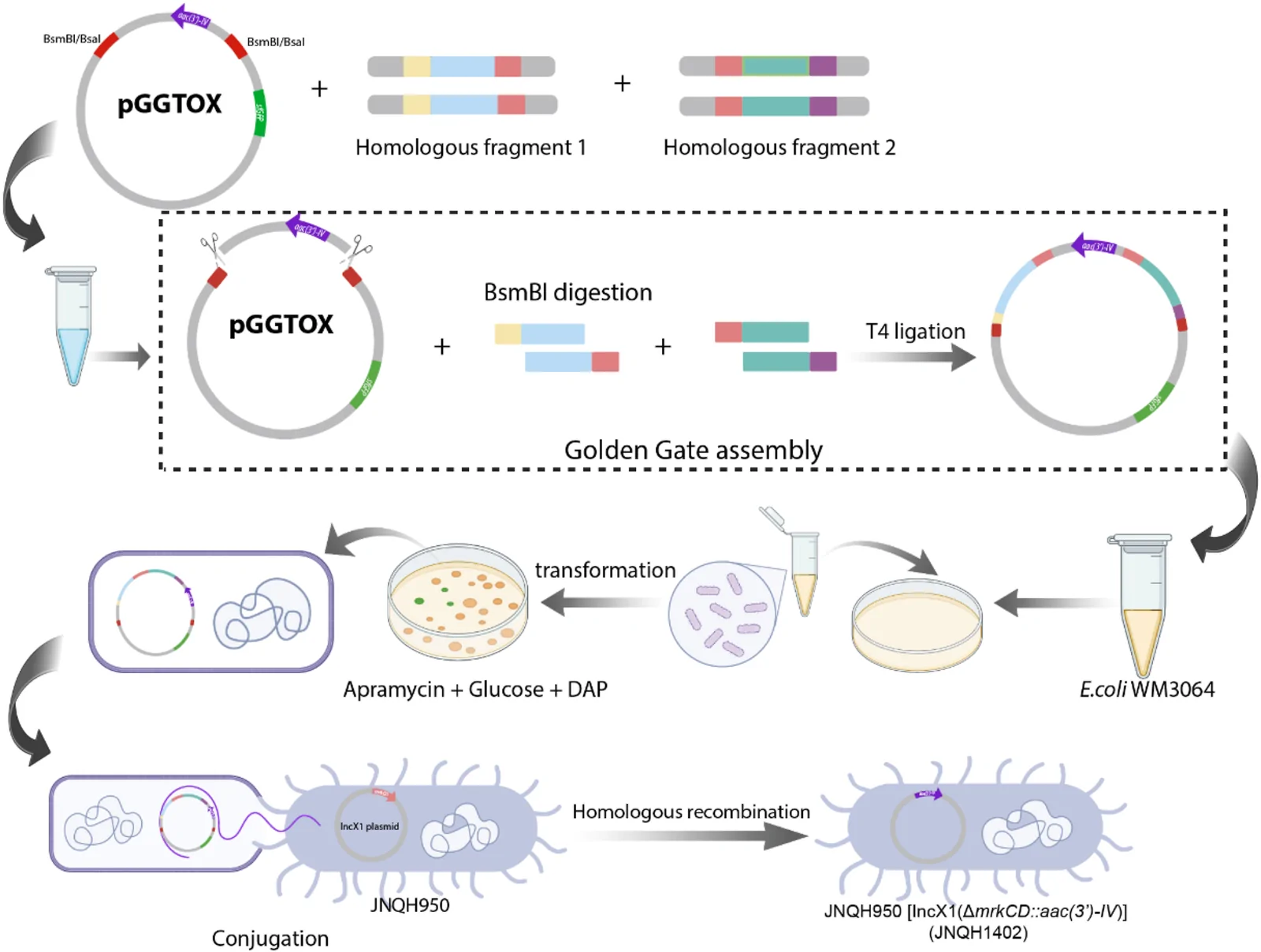
Strategy for insertion of an apramycin resistance cassette to disrupt the *mrkCD* genes.

To investigate the horizontal transfer characteristics of the *mrk*-carrying IncX1 plasmid, an apramycin resistance marker *aac(3’)-IV* was introduced using the our previously constructed pGGTOX system (Fig. 1) [21]. Two insertion sites were selected within the IncX1 plasmid: one located in a non-coding region, and the other within the *mrkCD* coding sequence, where the insertion simultaneously replaced the *mrkCD* genes.

Using the plasmid from strain JNQH950 as a template, homologous arm primers containing *Bsm*BI recognition sites were designed, and the flanking fragments of the insertion sites were amplified by PCR (Table S1). Following Golden Gate assembly [22] with pGGTOX plasmid, the amplified fragments were ligated to the backbone. The recombinant plasmid was subsequently transformed into competent *E.coli* WM3064 cells to construct donor strains. The donor strains were then mated with the recipient strain JNQH950, resulting in the acquisition of mutant strains carrying the apramycin resistance marker. The strains with the *aac(3’)-IV* inserted in the non-coding sequence and substituting *mrkCD* were designated as JNQH1304 and JNQH1402, respectively.

Using the plasmid from JNQH950 as a template, homologous arms flanking the *mrkCD* locus were amplified by PCR with the introduction of *Bsm*BI restriction sites. The pGGTOX plasmid, which carries an apramycin resistance (*aac(3’)-IV*) marker, was used as the assembly backbone. The amplified fragments were ligated into the backbone by Golden Gate assembly, and the recombinant plasmid was transformed into *E. coli* WM3064 to generate the donor strain. Conjugative transfer of the donor plasmid into the recipient strain, followed by induction of homologous recombination, yielded an apramycin-resistant mutant in which the *mrkCD* genes were deleted. The schematic workflow diagram was created using BioRender.

### 2.10 Conjugation assay

Conjugation experiments were performed using *E. coli* J53AziR as the recipient as previously described [23]. Briefly, the apramycin-resistant mutant strain JNQH1304 and the *mrkCD*-deletion mutant strain JNQH1402 were used as donor strains, while sodium azide–resistant *E. coli* J53 served as the recipient strain. Donor and recipient cells were cultured overnight at 37 °C. On the following day, both strains were grown in LB medium to the logarithmic phase, washed with PBS, and mixed at a 1:1 ratio. The mixture was applied to 0.45 μm filter paper, which was then placed on an LB agar plate, followed by overnight culture at 37 °C. The resulting bacterial lawn was resuspended and serially diluted, and aliquots were plated onto sodium azide (100 mg/L) and apramycin (30 mg/L) dual-selection agar plates to select for transconjugants. Conjugation frequency was calculated by dividing the number of transconjugants by the number of recipient cells. The highly conjugative IncN/FII plasmid characterized in our previous study was used as a positive control [15].

### 2.11 Growth curve analysis

The impact of the IncX1 plasmid on bacterial fitness was evaluated by monitoring growth in LB broth. Overnight cultures of each strain (JNQH950, JNQH1093, JNQH1100) were harvested, washed twice with phosphate-buffered saline (PBS), and resuspended in fresh LB medium to an initial optical density of 0.1 at 600 nm (OD_600_). Aliquots of 200 μL were transferred into sterile 96-well microtiter plates. Plates were incubated at 37 °C with continuous orbital shaking, and OD_600_ measurements were automatically recorded every 5 min for 24 h using a Multiskan™ FC Microplate Photometer (Thermo Scientific, IE). Four replicate growth curves were acquired for each strain.

### 2.12 Plasmid stability assay

Plasmid stability was assessed as previously described with minor modifications. Briefly, strains JNQH1304 (derived from JNQH950 and carrying the *aac(3’)-IV* gene) were inoculated separately into antibiotic-free LB broth and incubated overnight at 37 °C. Overnight cultures were serially passaged for 10 consecutive days (corresponding to 11 generations per day; 110 generations in total) by 1:1000 dilution into fresh antibiotic-free LB broth, followed by incubation at 37 °C with shaking at 200 rpm. On day 1, 3 and 5, a culture sample from each lineage was collected and preserved at −80 °C. On day 10, cultures were serially diluted and plated onto both apramycin-containing LB agar and antibiotic-free LB agar, followed by incubation at 37 °C for 16 h. Colony counts were used to calculate plasmid loss according to the formula:

$$\text{Plasmid}\;\text{loss}\;\text{rate}\;\left(\%\right)=\frac{\left(\text{Colony}\;\text{count}\;\text{on}\;\text{LB}\;\text{plates}\right)\;‐\;\left(\text{Colony}\;\text{count}\;\text{on}\;\text{apramycin}\;\text{plates}\right)}{\left(\text{Colony}\;\text{count}\;\text{on}\;\text{LB}\;\text{plates}\right)}\times 100\%$$

### 2.13 Scanning and transmission electron microscopy

For Scanning electron microscopy (SEM), overnight bacterial cultures were pelleted (2000 × g, 7 min), washed in PBS (pH 7.3), and fixed in 2.5% glutaraldehyde at 4 °C for 2 h. Samples were rinsed, post-fixed in osmium tetroxide, dehydrated through graded ethanol, and dried at the CO_2_ critical point. They were mounted on aluminum stubs with carbon tape, sputter-coated with 15 nm gold/palladium, and imaged by SEM. For transmission electron microscopy (TEM), bacteria were placed on carbon-coated copper grids for 3–5 min, stained with 2% phosphotungstic acid for 1–2 min, air-dried, and examined by TEM.

### 2.14 Bacterial survival under environmental stresses

To evaluate the role of *mrkABCDF* genes in bacterial tolerance to environmental stresses, we assessed changes in viable cell counts under acidic, hyperosmotic, oxidative, and desiccation conditions. Acid stress was applied using LB medium adjusted to pH 3.0 with HCl; hyperosmotic stress used LB supplemented with 8.0% (w/v) NaCl; oxidative stress used LB containing 2 mM H_2_O_2_. The strains tested included the wild type strain (JNQH950), an IncX1 plasmid-cured strain (JNQH1093), and a *Tn*6011 transposon knockout mutant (JNQH1100).

All strains were grown overnight on LB agar at 37 °C. Single colonies were inoculated into 5 mL LB broth and cultured at 37 °C with shaking (200 rpm) to mid-log phase. Cultures were adjusted to 0.5 McFarland (∼1 × 10^9^ CFU/mL), and 0.5 mL was transferred into 4.5 mL of the corresponding stress medium, followed by incubation at 37 °C with shaking at 180 rpm. At 0, 1, 2, and 3 h, 100 μL samples were collected. For acid and oxidative stress assays, samples were diluted or quenched with prechilled PBS or PBS containing 0.8% sodium thiosulfate, respectively; hyperosmotic samples were processed directly. Serial dilutions were plated on LB agar for colony enumeration. Survival curves were generated by plotting time (h) versus log_10_(CFU/mL), and differences among strains were used to assess the contribution of *mrk* gene cluster to stress responses [24].

To examine desiccation tolerance, overnight cultures of each strain were diluted 1:1000 and dispensed into U-bottom 96-well plates (50 μL per well). Plates were incubated in a humid chamber for 24 h and then uncovered to allow drying [25]. Subsequently, plates were transferred to a 37 °C incubator. At designated time points, 100 μL PBS was added to each well, mixed thoroughly, and the eluates were collected, serially diluted, and plated on LB agar to determine viable counts.

### 2.15 Strain labeling with sfGFP or luciferase using the Tn7 transposon system

To generate genetically stable reporter strains for longitudinal imaging, sfGFP or luciferase cassettes were integrated as single copies at the attTn7 site downstream of *glmS* using an established Tn7 site-specific transposition system. This approach was selected because it enables insertion at a defined chromosomal locus without requiring strain-specific homologous recombination arms [26,27]. The detailed procedure is provided in Supplementary file and Fig. S1. ALL plasmids and primers are detailed in Table 1 and Table S1.

### 2.16 Phagocytosis assessment by RAW264.7 macrophages

Murine macrophage-like RAW264.7 macrophages were maintained in RPMI 1640 medium supplemented with 10% fetal bovine serum at 37 °C in a 5% CO_2_ atmosphere until cultures reached approximately 50–60% confluence. Wild-type JNQH950 and the *Tn*6011-deletion mutant JNQH1100, both labeled with *sfGFP,* on the chromosome were grown to logarithmic phase, adjusted to a turbidity equivalent to a 0.5 McFarland standard, and resuspended in fresh culture medium. The bacterial suspensions were added to RAW264.7 cells at a multiplicity of infection (MOI) of 20:1.

At 1, 3, 6, and 18 h post-infection, the culture medium was removed and cells were washed with medium containing ceftazidime (16 μg/mL) to eliminate extracellular bacteria. Phagocytosis and intracellular bacterial localization were subsequently examined using fluorescence microscopy. All experiments were performed in triplicate on independent occasions.

### 2.17 Antibiotic-induced biofilm formation assay

The effects of ceftazidime (CAZ), levofloxacin (LVX), and meropenem (MEM) on *mrk*-mediated biofilm formation were evaluated. Minimum inhibitory concentrations (MICs) for the wild-type strain (JNQH950), the *Tn*6011 knockout (JNQH1100), and the IncX1-cured mutant (JNQH1093) strains were determined by broth microdilution. Overnight cultures of each strain were diluted 1:1000, and sub-MIC antibiotic solutions were prepared in two-fold serial dilutions. Diluted bacterial suspensions were co-incubated with the respective antibiotic concentrations in humid chambers overnight. Biofilm formation was quantified the following day using crystal violet staining as described above. All assays were performed in triplicate.

### *2.*18 RT-qPCR of *mrk* gene clusters induced by antibiotics

Overnight bacterial cultures grown in LB were diluted 1:1000 into LB supplemented with the same antibiotic concentrations used in the biofilm assay and incubated at 37 °C with shaking at 200 rpm. At an OD600 of 0.4–0.6, the cultures were immediately placed on ice and washed twice with RNase-free PBS. Total RNA was extracted using the EasyPure® RNA Kit (TransGen Biotech), and cDNA was synthesized using TransScript® Uni All-in-One First-Strand cDNA Synthesis SuperMix. RT-qPCR was performed using PerfectStart® Green qPCR SuperMix on an Applied Biosystems™ QuantStudio™ 5 Real-Time PCR System. RNA input, reaction mixtures, and reverse-transcription and amplification programs followed the manufacturers' instructions. Relative expression was calculated using the 2^−ΔΔCt^ method, with *gyrA* as the reference gene and untreated LB cultures as the calibrator. Three independent biological replicates containing *gyrA* measurements were analyzed. Statistical analyses were performed on ΔCt values using two-sided paired Student's *t*-tests. Unadjusted *P* < 0.05 was considered nominally significant. Benjamini-Hochberg-adjusted q < 0.05 was considered significant after correction for multiple comparisons.

### 2.19 Mouse intestinal colonization assay

The luciferase-encoding gene was integrated into the chromosomal genome of the experimental strains by *Tn*7 site-specific integration as described above. Four-week-old BALB/c female mice were randomly assigned to two groups (n = 6 per group) and orally inoculated with either the luciferase-labeled wild-type strain (JNQH950) or the luciferase-labeled *Tn*6011 deletion mutant (JNQH1100). Prior to infection, mice were provided streptomycin-containing drinking water (5 g/L) for 24 h and fasted overnight. Strains were grown overnight, subcultured to mid-logarithmic phase (0.5 McFarland), washed with PBS, and prepared for gavage. For each inoculation, 1 × 10^7^ CFU of bacteria were mixed with 100 μL of 20% (w/v) sucrose and 6% (w/v) NaHCO_3_ and administered orally using a gavage needle. Mice were gavaged once daily for three consecutive days.

For in vivo imaging, mice were anesthetized with isoflurane, injected intraperitoneally with the luciferase substrate, and bioluminescence signals were captured using an IVIS Spectrum imaging system (PerkinElmer). Imaging was performed daily for five consecutive days to monitor intestinal colonization dynamics.

### 2.20 Whole-genome sequencing analysis

Whole-genome sequencing was performed for the mutant strains of JNQH1093, JNQH1100, JNQH1304, JNQH1402 (Table 1). To verify the intended genetic modifications and assess potential off-target mutations, sequencing reads from each mutant strain were compared with the genome sequence of the wild-type strain JNQH950. Sequence variants were identified using breseq v0.40.1 [28] with default parameters. Candidate variants were manually inspected to confirm the designed mutations and to exclude additional unintended mutations.

### 2.21 Statistical analysis

To assess bacterial survival dynamics under environmental stresses, including desiccation, acid, hypertonic, and oxidative stress, death rates were estimated for each independent biological replicate of each strain. Viable cell counts were expressed as log_10_(CFU/mL), and a linear regression model was fitted against time:

log_10_(CFU/mL) = *β*_*0*_ + *β*_1_(Time) + ε

The decay rate, κ, was defined as the negative slope of the fitted model, κ = -β1, representing the loss of log_10_(CFU/mL) per hour or day. For the desiccation, hypertonic stress, and oxidative stress assays, slopes were calculated across the complete multi-point time course. For the acid tolerance assay, because viable counts in highly susceptible strains dropped below the limit of detection by 2 h, the decay rate was calculated from the initial decline phase from 0 to 1 h.

Differences in estimated decay rates among strains were evaluated using one-way analysis of variance (ANOVA). Post hoc comparisons between the wild-type strain JNQH950 and each mutant or complemented strain were performed using Dunnett's multiple-comparison test.

All experimental data were expressed as mean ± SD, with group differences assessed by Student's *t*-test. For growth curves, μ_max_ was calculated separately for each replicate growth curve as the maximum ordinary least-squares slope of ln(OD_600_) versus time in a fixed 13-measurement (approximately 1-h) rolling window. The derivative strains were each compared with the WT JNQH950 using two-sided Welch's *t*-tests. Statistical significance defined as *P* < 0.05; analyses and figure preparation were performed using R v4.5.3 or GraphPad Prism v10.1.2.

## 3 Results

### 3.1 Genomic organization and sequence conservation of the IncX1-borne mrkABCDF locus

**Fig. 2:**
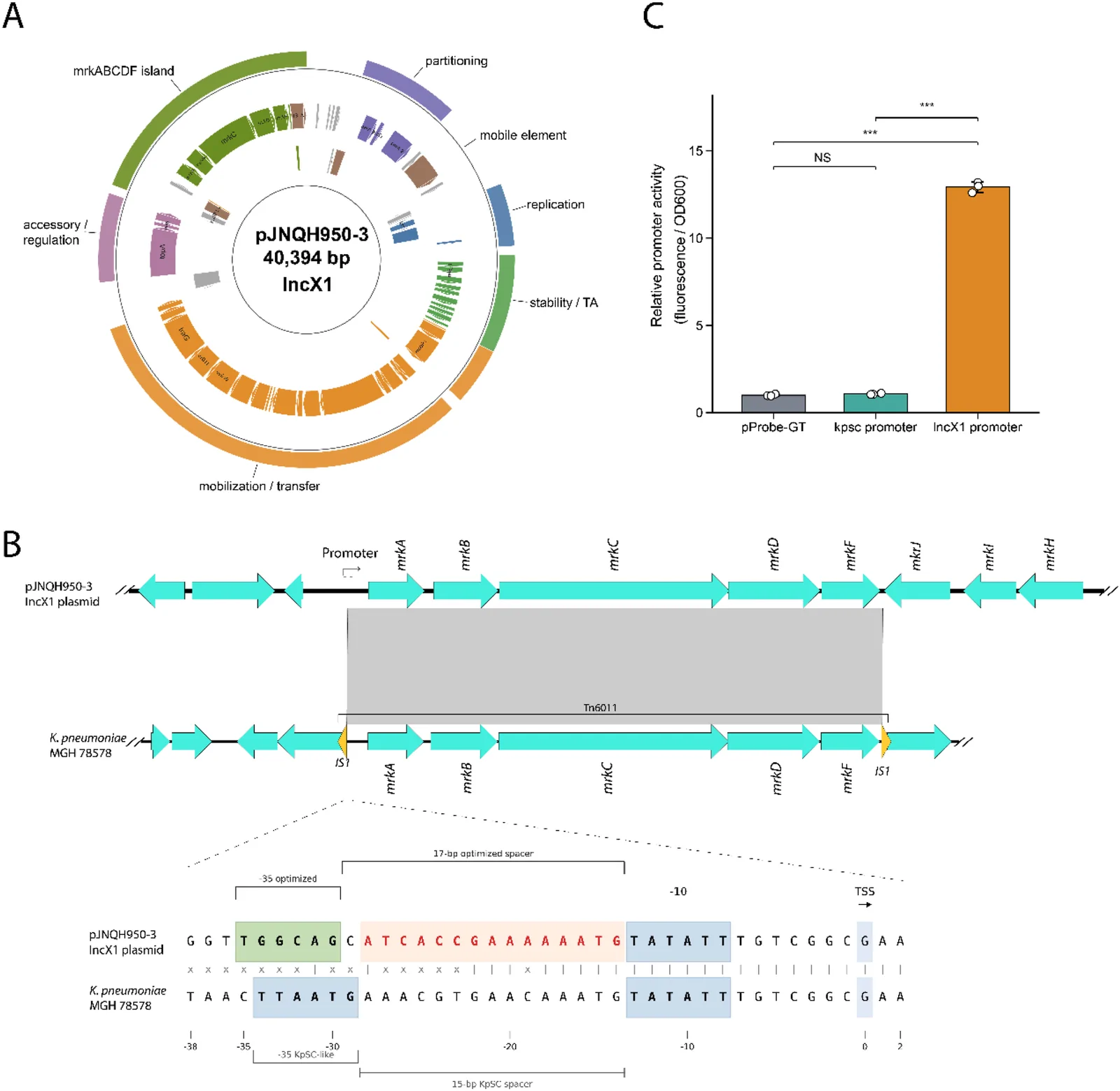
Genomic architecture of the IncX1 plasmid pJNQH950-3 and comparative promoter activity of the *mrkABCDF* locus. (A) Circular map of pJNQH950-3. The 40,394-bp IncX1 plasmid contains the IncX1 replication/iteron region, stability/toxin-antitoxin region, oriT/mobP1 mobilization region, type IV secretion/conjugative transfer region, topA/hha region, and the IS1-flanked *mrkABCDF* island. (B) Genetic organization of the *Salmonella* Thompson IncX1-borne *mrk* cluster compared with the KpSC archetype. The *Salmonella* pJNQH950-3 locus contains *mrkABCDF* flanked by IS1-like elements and an upstream IS1-associated putative promoter/TSS. The reference locus from *K. pneumoniae* MGH 78578 contains the conserved *mrkABCDF* structural genes plus the adjacent *mrkHIJ* regulatory module. Grey lines connect orthologous structural genes. Red bases mark the IS1-associated, plasmid-like promoter spacer in pJNQH950-3. Blue/green boxes mark candidate - 35/-10 elements. (C) Promoter activity measured using sfGFP reporter. Green fluorescence intensity was normalized to OD_600_ for each replicate and expressed relative to control activity. Circles represent individual replicate measurements (n = 3). (For interpretation of the references to colour in this figure legend, the reader is referred to the Web version of this article.)

The 40,394-bp IncX1 plasmid pJNQH950-3 exhibits a typical IncX1 backbone organization, incorporating replication and stability modules, mobilization and conjugative transfer regions, and a *mrkABCDF* gene cluster (Fig. 2A). The *mrkABCDF* locus preserved the same gene order as the KpSC operon but was embedded within an IS1-associated region corresponding to the mobile transposon Tn*6011*. Unlike the KpSC locus, which contains the adjacent *mrkHIJ* regulatory module, the complete pJNQH950-3 sequence lacked homologues of *mrkH, mrkI and mrkJ* (Fig. 2B).

The structural proteins were highly conserved between the *Salmonella* IncX1-borne locus and the KpSC reference. Pairwise amino-acid identities were 99.5% for MrkA, 96.7% for MrkB, 100% for MrkC, 97.6% for MrkD and 99.5% for MrkF. MrkD contained eight amino-acid substitutions relative to the KpSC sequence, with the greatest local divergence occurring in the central region of the protein.

The putative promoter upstream of *mrkA* in the IncX1-borne locus was located within the IS1-associated region. Both sequences contained a similar putative −10 element, whereas their putative −35 regions differed (Fig. 2B).

### 3.2 The IncX1-borne mrkA promoter exhibits enhanced activity

After normalization to OD_600_ and expression relative to the promoterless pPROBE-GT control, the lncX1 promoter showed a relative activity of 12.93 ± 0.30, compared with 1.07 ± 0.04 for the KpSC promoter. In contrast, fluorescence from the KpSC construct did not differ significantly from that of the promoterless vector. Thus, the IncX1 promoter displayed more than 12-fold higher activity than the KpSC promoter (Fig. 2C).

### 3.3 CRISPR-Cas9 editing enables mrk cluster excision or IncX1 plasmid loss

**Fig. 3:**
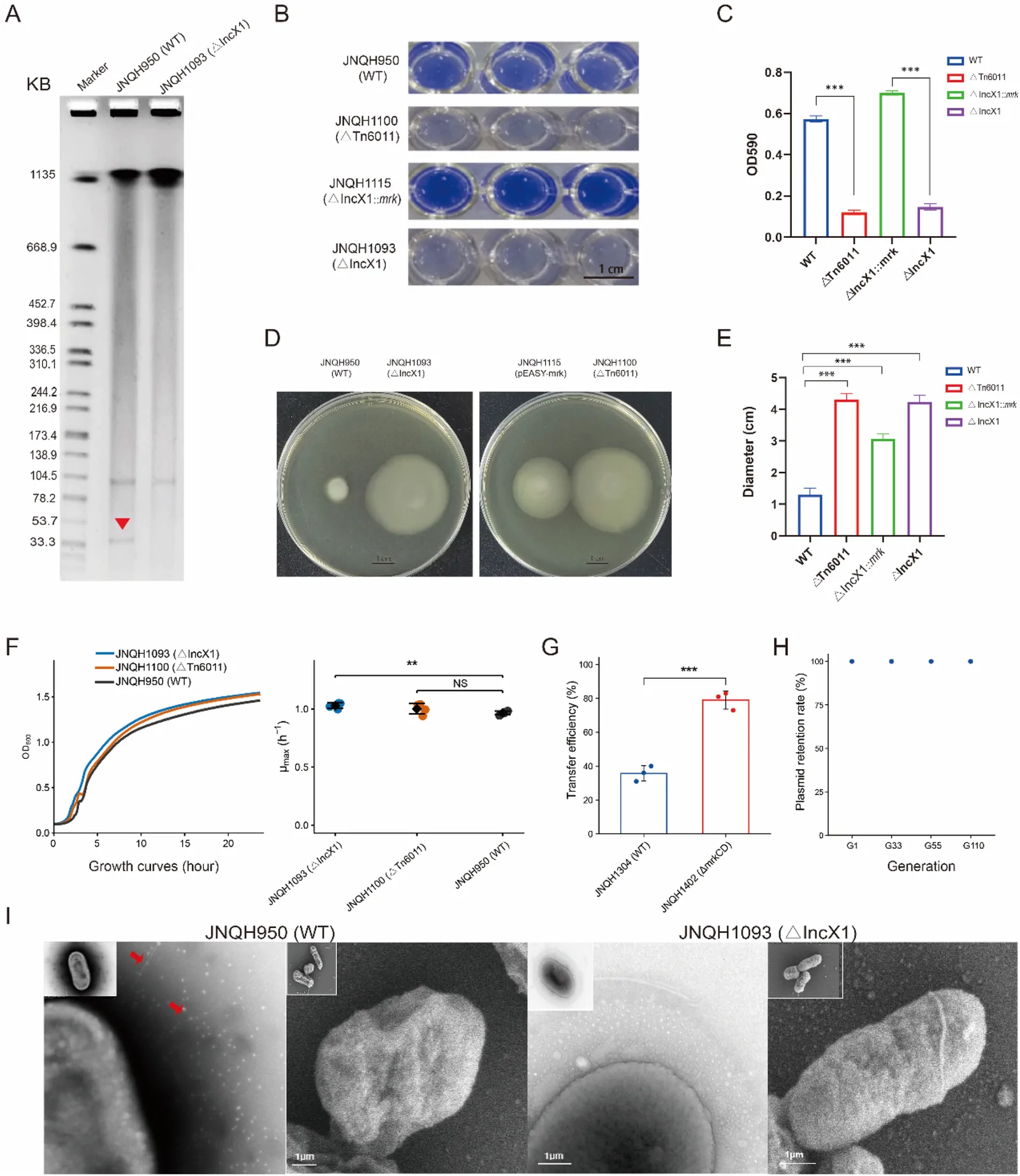
Functional characterization of the IncX1 plasmid and the *mrk* gene cluster. (A) S1-PFGE analysis confirmed successful curing of the IncX1 plasmid; the red arrow indicates the IncX1 plasmid carrying the *mrkABCDF* operon. (B–C) Quantification of biofilm formation in wild-type (WT), *mrk* knockout (Δ*mrkABCDF*), and complemented strains (ΔIncX1*mrk*). Biofilm biomass was measured using crystal violet staining. Data represent three replicates. (D–E) Swimming motility of WT, Δ*mrk*, and *mrk* complemented strains assessed on 0.3% agar plates. Halo diameters represent three replicates. (F) Growth curves and replicate-specific μ_max_ values were determined. Curves represent three replicates. For each replicate growth curve, μ_max_ was calculated as the maximum ordinary least-squares slope of ln(OD_600_) versus time across a fixed 13-measurement (approximately 1-h) rolling window. Points represent individual replicate growth curves; diamonds and error bars denote mean ± SD. Each derivative was compared with JNQH950 using a two-sided Welch's *t*-test. (G) Conjugation frequencies of WT, Δ*mrk*, and *mrk* complemented strains. Data represent three replicates. (H) Plasmid stability of the IncX1 plasmid over 110 generations without antibiotic selection. Data represent three replicates. (I) Representative TEM and SEM micrographs of the wild-type (WT) and *mrk* knockout (Δ*mrk*) strains demonstrate abundant fimbrial structures in the WT strain, which were absent in the Δ*mrk* mutant. Data are presented as mean ± SD; statistical significance was determined by Student's *t*-test. NS: not significant, *P < 0.05, **P < 0.01, ***P < 0.001. (For interpretation of the references to colour in this figure legend, the reader is referred to the Web version of this article.)

Using CRISPR-Cas9–mediated genome editing, we successfully knocked out the *mrk* gene cluster and simultaneously obtained mutants lacking the IncX1 plasmid. Following plasmid curing with the pCasCure system, ten single colonies were randomly selected for PCR verification. All isolates showed complete deletion of the *mrkA* gene. However, one clone retained the IncX1 replicon. PCR amplification of the regions flanking *Tn*6011 followed by Sanger sequencing confirmed that the transposon was fully excised in the IncX1-positive mutants. S1-PFGE analysis demonstrated that the IncX1 plasmid was completely cured (Fig. 3A).

### 3.4 The mrk gene cluster enhances biofilm formation and is associated with reduced swimming motility

Crystal violet assays demonstrated that the wild-type strain exhibited robust biofilm formation, whereas all *mrk* cluster deletion mutants (including the ΔIncX1 and the Δ*Tn*6011 mutant) showed markedly reduced biofilm biomass. Restoration of the *mrk* gene cluster rescued biofilm formation, supporting its role in this phenotype (Fig. 3B and C).

Soft-agar motility assays showed that deletion of either the IncX1 plasmid or the *mrk-*carrying *Tn*6011 transposon resulted in substantially larger swimming halos compared with the wild-type strain, indicating a pronounced increase in swimming motility in both mutants. Complementation with the *mrkABCDF* gene cluster partially reduced halo size, but motility did not return to wild-type levels, demonstrating that the complemented strain remained more motile than the wild type (Fig. 3D and E).

### 3.5 High conjugation efficiency of mrk-harboring IncX1 plasmids

Conjugation assays showed efficient transfer of the IncX1 plasmid in both strains. The transfer efficiency was 35.7 ± 4.5% in JNQH1304 and increased to 79.0 ± 5.3% in JNQH1402 (Δ*mrkCD*), representing an approximately 2.2-fold increase (Fig. 3G). These results suggest that deletion of *mrkCD* enhances plasmid transfer.

### 3.6 Fitness cost assessment of IncX1 plasmid

We assessed in vitro growth capacity of the IncX1 derivatives by calculating μ_max_ for each replicate growth curve. JNQH1093 (ΔIncX1) had a higher μ_max_ than the WT JNQH950 (*P* = 0.0068). JNQH1100 (Δ*Tn*6011) had a μ_max_ of 1.002 ± 0.046 h^−1^ and did not differ significantly from JNQH950 (*P* = 0.2192). Thus, loss of the complete IncX1 plasmid increased the maximum growth rate under the tested LB conditions, whereas deletion of the *Tn*6011 region alone did not produce a statistically significant effect (Fig. 3F).

### *3.7* *IncX1 plasmid stability in Salmonella* Thompson *JNQH950*

Plasmid stability was evaluated in strain JNQH1304 over 110 generations of serial passage in antibiotic-free LB broth. After 10 consecutive days of growth, the proportion of colonies retaining the IncX1 plasmid remained consistently high across all lineages, with no significant reduction observed during the experiment. On day 10, colony counts on apramycin plates were nearly identical to those on antibiotic-free plates, corresponding to a plasmid loss rate close to 0%. These findings demonstrated that the *mrkABCDF* carrying IncX1 plasmid was stably maintained under non-selective conditions throughout the experimental period (Fig. 3H).

### 3.8 Type 3 fimbriae detected in wild type but absent in IncX1 cured strain

SEM revealed no obvious differences in overall cell surface morphology in the *IncX1 cured* strain JNQH950. In contrast, TEM of the wild-type strain JNQH950 identified surface filaments measuring approximately 3–10 μm, consistent with type 3 fimbriae. These structures were absent in the *mrk* knockout strain (Fig. 3I).

### 3.9 Mrk gene cluster enhances Salmonella survival under environmental stresses

**Fig. 4:**
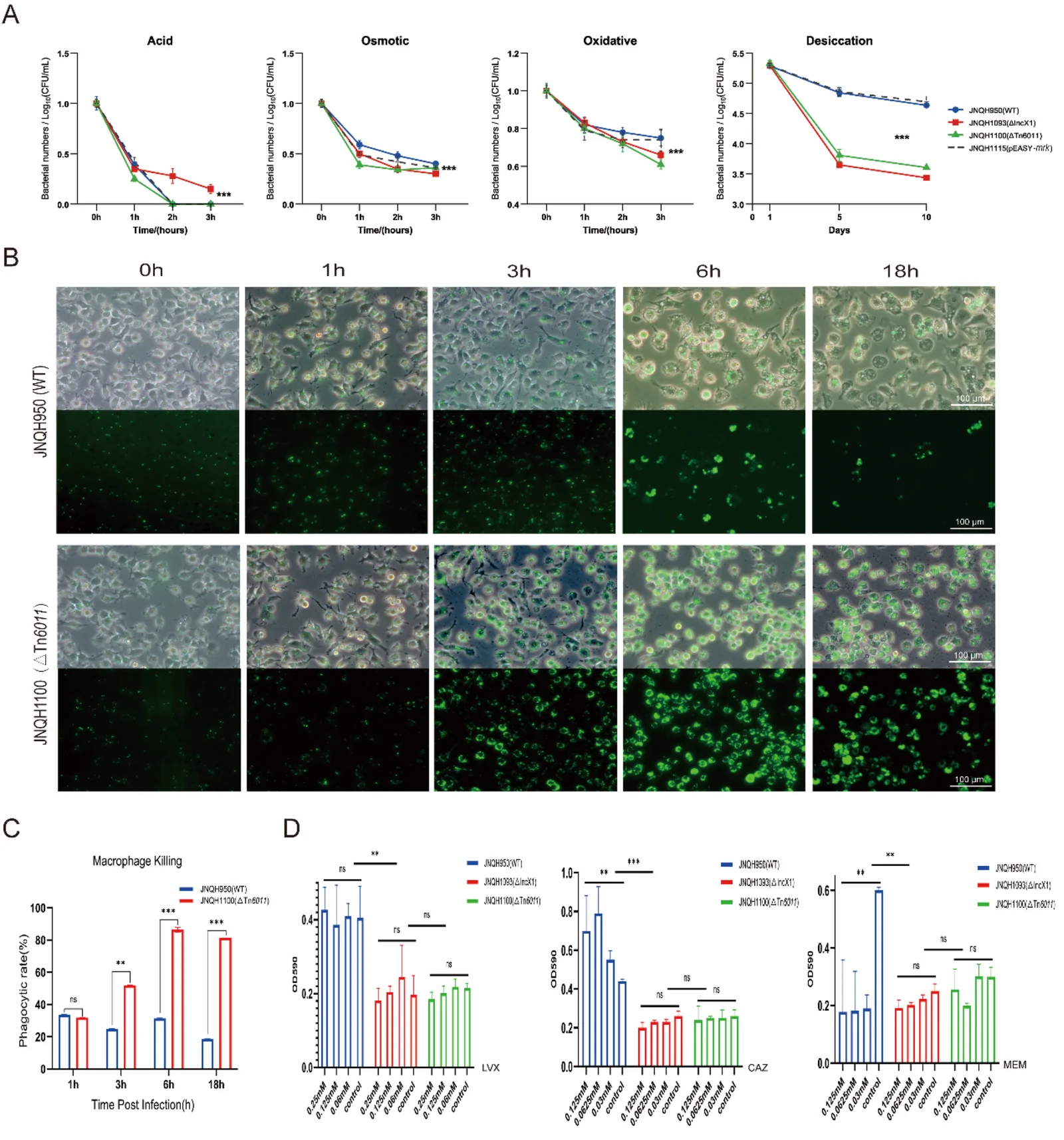
Stress responses, phagocytosis, and antibiotic effects on *mrk-*dependent biofilm formation. (A) Environmental stress survival kinetics. Survival of JNQH950 (WT), JNQH1093 (ΔIncX1), JNQH1100 (ΔTn6011), and the *mrkABCDF* complemented strain JNQH1115 (pEASY-*mrkABCDF*) under acid, osmotic, oxidative, and desiccation stress. Data are shown as mean ± SD of three replicates. Significance was determined by one-way ANOVA followed by Dunnett's test comparing decay rates with WT; ***P < 0.001. (B) Phagocytosis of wild-type and *mrk-*deleted strains by RAW264.7 macrophages at different time points. Scale bars are displayed at the bottom of each figure panel. (C) Wild-type *Salmonella* strain JNQH950 exhibited greater resistance to macrophage phagocytosis over prolonged incubation time. (D) Antibiotic effects on *mrk-*mediated biofilm formation. Low concentrations of ceftazidime induced biofilm formation, levofloxacin showed no effect, whereas meropenem markedly inhibited biofilm formation even at low concentrations. Data represent mean ± SD from three independent experiments; statistical significance was determined by Student's *t*-test. Ns: not significant, *P < 0.05, **P < 0.01, ***P < 0.001.

Decay-rate analysis showed that loss of the IncX1 plasmid or disruption of the *mrk*-containing region altered *Salmonella* survival in a stress-dependent manner. Under desiccation stress, both JNQH1093 and JNQH1100 showed significantly higher decay rates than WT, whereas the complemented strain JNQH1115 did not differ from WT. This indicates that the *mrk* gene cluster contributes strongly to survival during prolonged desiccation (Fig. 4A).

A similar pattern was observed under oxidative stress, where JNQH1093 and JNQH1100 showed significantly increased decay rates compared with WT, while JNQH1115 remained comparable to WT. In contrast, the acid-stress phenotype was less consistent: only JNQH1100 showed a significantly increased initial decay rate, whereas JNQH1093 and JNQH1115 were not significantly different from WT (Fig. 4A).

Under hypertonic stress, JNQH1093 showed a significantly higher decay rate than WT, whereas JNQH1100 was not significantly different and JNQH1115 showed only a marginal difference after correction (Fig. 4A). This suggests that hypertonic-stress survival may involve additional IncX1 plasmid-associated factors beyond the *mrk* gene cluster. Together, these results support a prominent role for the *mrk* gene cluster in desiccation resistance, with additional contributions to oxidative-stress survival, while its role in acid and hypertonic stress responses appears more limited or context dependent.

### 3.10 Mrk gene cluster confer resistance to macrophage phagocytosis

RAW264.7 macrophage assays revealed that, at 1 h post-infection, both wild-type (JNQH950) and *mrk-*deficient strain (JNQH1100) induced morphological changes in host cells, including pseudopodia extension and increased membrane ruffling. Fluorescence microscopy showed scattered bacterial distribution at this stage, and phagocytosis levels were comparable between strains (Fig. 4B and C). By 3 h, however, intracellular uptake of the *mrk-*deficient strain was more pronounced than that of the wild type. At 6 h and 18 h, this difference became increasingly evident, indicating that strains harboring the *mrk* gene cluster exhibited increased resistance to macrophage phagocytosis.

### 3.11 Carbapenems effectively suppress mrkABCDF-mediated biofilm formation

We compared biofilm formation of the wild-type strain (JNQH950), the *Tn*6011 knockout (JNQH1100), and the IncX1-cured mutant (JNQH1093) under antibiotic stress. The MICs of ceftazidime, meropenem, and levofloxacin were 0.25 μg/mL, 0.25 μg/mL, and 0.5 μg/mL, respectively. Interestingly, these antibiotics exhibited distinct effects on *mrk-*mediated biofilm formation. Levofloxacin did not significantly alter biofilm production at 0.25–0.06 μg/mL. In contrast, ceftazidime promoted biofilm formation at 0.125–0.03 μg/mL, with stronger enhancement at higher concentrations, whereas meropenem inhibited biofilm formation over the same concentration range. These findings highlight the potent anti-biofilm activity of carbapenems against *mrk-*mediated biofilm development.

RT-qPCR revealed non-uniform regulation of the *mrk* genes. Levofloxacin at 0.06 μg/mL reduced *mrkD* expression to 0.39-fold (*P* = 0.006), whereas the 3.12-fold increase in *mrkA* was not nominally significant (*P* = 0.078). At 0.125 μg/mL, levofloxacin increased *mrkA* expression 3.12-fold (*P* = 0.021) while the 1.53-fold increase in *mrkF* was not significant (*P* = 0.187). Ceftazidime at 0.03 μg/mL increased *mrkA*, *mrkB*, *mrkC*, and *mrkD* expression by 5.97-fold (*P* = 0.038), 3.20-fold (*P* = 0.076), 2.59-fold (*P* = 0.017), and 1.78-fold (*P* = 0.063), respectively. Meropenem at 0.0625 μg/mL increased *mrkC*, *mrkD*, and *mrkF* expression by 2.45-fold (*P* = 0.038), 1.82-fold (*P* = 0.173), and 1.76-fold (*P* = 0.134), respectively. At 0.125 μg/mL, meropenem reduced *mrkC*, *mrkD*, and *mrkF* expression to 0.41-fold (*P* = 0.013), 0.24-fold (*P* = 0.033), and 0.30-fold (*P* = 0.052), respectively (Fig. S2). However, none remained significant after Benjamini–Hochberg correction (Table S2). Therefore, these findings are interpreted as concentration-dependent transcriptional trends rather than definitive statistically significant changes.

### 3.12 Mrk gene cluster enhances intestinal colonization and systemic dissemination in vivo

**Fig. 5:**
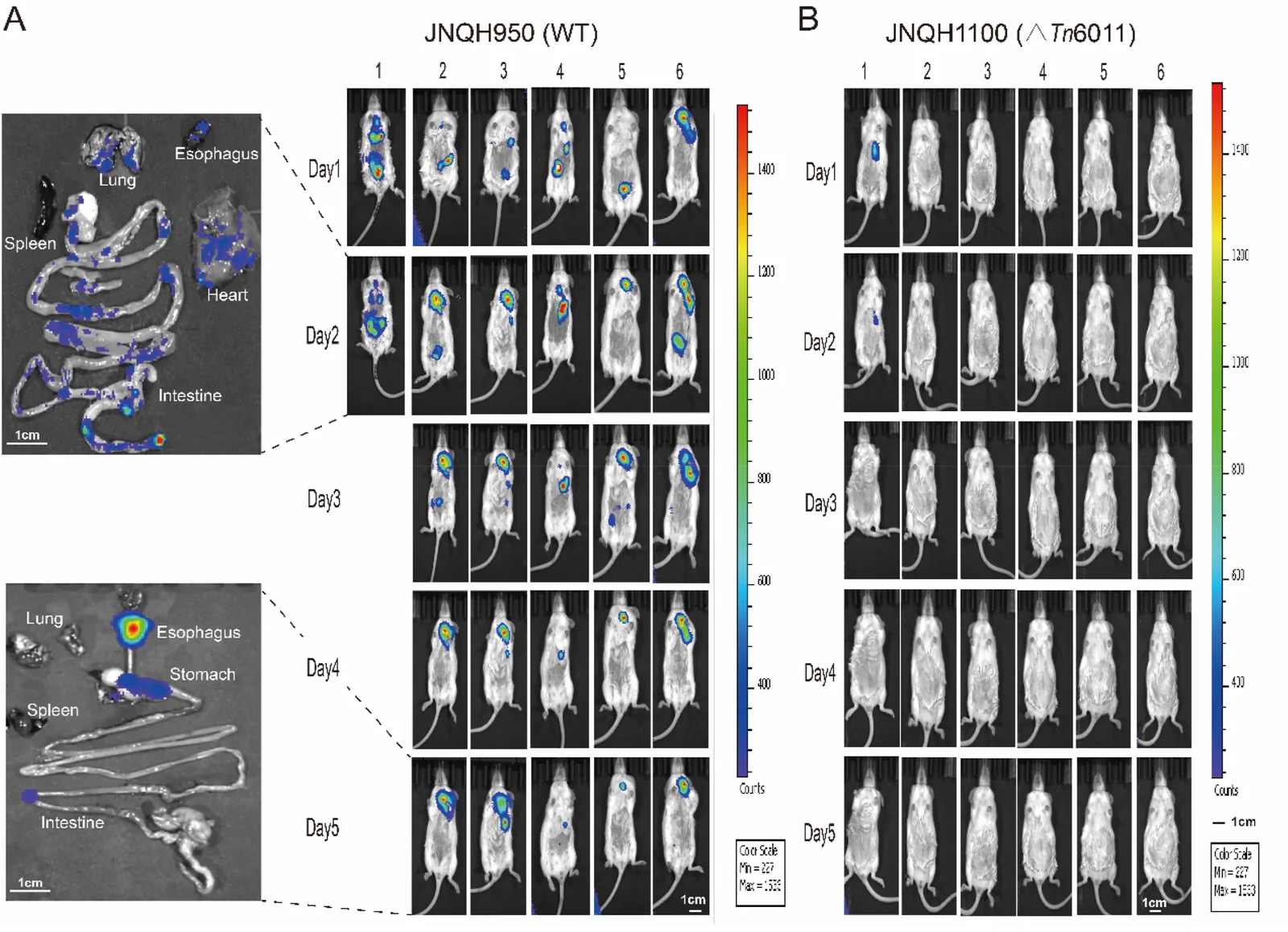
*Mrk* gene cluster drive intestinal colonization and tissue invasion by *Salmonella*. Bioluminescence imaging of *luciferase* labeled *Salmonella* strains following oral infection in mice. Wild-type strains carrying the *mrk* cluster showed strong colonization and dissemination, with signals detected in the esophagus, colon, and terminal ileum. Organ biopsies confirmed the corresponding luminance patterns (A). Colonization was markedly attenuated in mice infected with the *mrkABCDF* knockout mutant (B). Scale bars are displayed at the bottom of the figure panels.

In vivo infection experiments demonstrated a strong association between the *mrk* gene cluster and the capacity of *Salmonella* to colonize the gastrointestinal tract and invade host tissues. Following oral inoculation, mice infected with the wild-type strain exhibited substantial intestinal colonization, with the esophagus identified as the predominant site of bacterial recovery. Notably, systemic dissemination was observed in one mouse, as evidenced by bacterial isolation from multiple extraintestinal organs, including the lung and heart, indicating invasive potential (Fig. 5).

In contrast, colonization was markedly attenuated in mice infected with the *mrkABCDF* knockout mutant. Only one mouse showed detectable bacterial colonization at day 1 post-inoculation, and no evidence of systemic spread was observed. These findings indicate that the *mrk* gene cluster plays a critical role in promoting gastrointestinal colonization and facilitating invasive dissemination of *Salmonella* in vivo.

### 3.13 Genome verification of mutant strains

To confirm the genetic integrity of the mutant strains, we performed whole-genome sequencing of all mutants and compared their sequences with that of the wild-type strain JNQH950. In each mutant, the designed mutation was correctly detected at the expected genomic locus. No additional single-nucleotide variants, insertions, deletions, or other detectable off-target mutations were observed relative to the wild-type strain JNQH950 under the applied variant-calling criteria. These results confirmed that the phenotypes observed in the mutant strains were attributable to the intended genetic modifications.

## 4 Discussion

This study demonstrates that the plasmid-encoded *mrkABCDF* cluster is a major contributor to biofilm formation and virulence-associated phenotypes in *Salmonella* Thompson. The presence of *mrk* genes markedly increased biofilm biomass, enhanced tolerance to environmental stresses, and promoted persistence in host tissues. The atypical dissemination of *mrk*-positive strains to the skin site suggests that type 3 fimbriae may expand the pathogenic potential of *Salmonella* beyond classical intestinal colonization [29].

Type 3 fimbriae are well-recognized adhesins across *Enterobacteriaceae* [30,31], and our findings align with extensive work in KpSC, where *mrkABCDF* is chromosomally encoded and strongly associated with biofilm formation and epithelial adherence [32]. In KpSC, type 3 fimbriae support respiratory persistence by promoting attachment to airway cells and resisting clearance mechanisms. However, the regulatory context differs markedly: whereas KpSC relies on the c-di-GMP-dependent MrkH/MrkJ system [33], plasmid-borne *mrk* clusters—such as those in JNQH950—are driven by an IS1-associated promoter, enabling strong expression independent of MrkH. This regulatory rewiring likely contributes to the heightened biofilm phenotype observed in *Salmonella*.

The IncX1 plasmid pJNQH950-3 exhibits exceptionally high conjugation efficiency and exemplifies the role of IncX1 plasmids as efficient mobile backbones within *Enterobacteriaceae* [13]. Its conserved architecture—comprising the replication/iteron module, stability and toxin–antitoxin systems, the *oriT/mobP1* mobilization region, and a complete type IV secretion system—provides the structural basis for robust horizontal transfer [34,35]. Notably, deletion of *mrk* gene cluster markedly increased plasmid transfer frequency to nearly 80%. This enhancement likely reflects the loss of type 3 fimbriae, which normally extend from the cell surface and may impose steric hindrance that limits the intimate cell–cell contact required for conjugation. Thus, while the *mrkABCDF* cluster promotes strong adhesion and biofilm formation, it simultaneously constrains plasmid transmissibility, revealing a trade-off between surface adherence and conjugative efficiency.

Although IncX1 plasmids do not universally encode antimicrobial resistance (AMR) genes, several studies have reported IncX1 plasmids carrying *qnrS1*, *bla*_TEM_, and other AMR determinants [36,37]. Importantly, plasmids harboring both AMR genes and *mrk* clusters have been described, creating a potential route for the convergence of adhesion-associated traits and drug resistance in enteric bacteria [35]. This underscores the evolutionary flexibility of the IncX1 incompatibility group and its ability to accumulate adaptive modules beyond core conjugation functions.

In pJNQH950-3, the *mrkABCDF* locus resides on a Tn6011 transposon rather than the chromosome. The IS1-embedded promoter likely drives expression in a manner that bypasses host regulatory constraints, potentially amplifying adhesion, biofilm formation, and stress-resistance phenotypes. Such promoter rewiring may accelerate the emergence of biofilm-adapted *Salmonella* lineages with enhanced environmental fitness.

Antibiotic exposure produced distinct effects on biofilm formation. Sub-inhibitory ceftazidime increased *mrk*-dependent biofilm formation, levofloxacin had minimal impact, and meropenem strongly inhibited biofilm development even below MIC levels. The anti-biofilm activity of meropenem may involve interference with fimbrial assembly, efflux functions, or virulence-associated pathways [38]. RT-qPCR results only partially explained these phenotypes, suggesting additional layers of regulation—including growth dynamics, extracellular matrix production, stress-response pathways, post-transcriptional control, and fimbrial assembly—shape the biofilm response [[39], [40], [41]]. Further transcriptomic and genetic analyses will be required to define how antibiotic exposure integrates with *mrk*-dependent and *mrk*-independent biofilm pathways.

A clear trade-off between *mrk*-associated biofilm formation and swimming motility was observed in the JNQH950 background. While *mrk* enhanced adhesion and biofilm formation, it reduced motility, and complementation did not fully restore wild-type swimming. Given the diverse fimbrial repertoire encoded by *Salmonella* genomes, the heterologous *mrk* cluster may interact with endogenous fimbrial or flagellar regulatory networks [42], altering the balance between motility and surface-associated growth. The IncX1 plasmid also imposed a measurable fitness cost yet remained stable without antibiotic selection, suggesting context-dependent benefits of enhanced adhesion.

In vivo colonization patterns further support a fimbrial-dependent adhesion phenotype. Most mice inoculated with the wild-type strain exhibited modest colonization of the terminal ileum, consistent with the well-established route of *Salmonella* entry through ileal Peyer's patches. One mouse developed extensive ileal colonization followed by rapid systemic dissemination and death, a pattern characteristic of Peyer's patch–mediated invasion reported in other *Salmonella* studies [43]. These findings suggest that *mrkABCDF*-mediated adhesion may increase opportunities for translocation across intestinal lymphoid tissues, warranting further investigation into how plasmid-encoded type 3 fimbriae influence host entry routes and systemic spread.

Overall, this study provides compelling evidence that plasmid-encoded *mrkABCDF* genes substantially enhance biofilm formation, environmental persistence, and tissue invasiveness in non-typhoidal *Salmonella* Thompson. By promoting intestinal colonization and prolonged shedding, *mrk*-positive strains may increase the risk of environmental contamination, particularly of water sources. These findings highlight the importance of monitoring *mrk*-carrying plasmids and inform future strategies for preventing and managing *Salmonella* infections.

## CRediT authorship contribution statement

**Ke Liu:** Visualization, Validation, Methodology, Formal analysis. **Liya Feng:** Visualization, Validation, Methodology, Data curation. **Lu Ouyang:** Resources, Methodology. **Xinpeng Li:** Visualization, Methodology. **Xiaohong Shi:** Supervision, Project administration. **Liang Chen:** Writing – review & editing. **Mingju Hao:** Writing – review & editing, Writing – original draft, Supervision, Resources, Funding acquisition, Formal analysis, Conceptualization.

## Ethics of experimentation

The study was conducted in accordance with the Declaration of Helsinki and approved by the Institutional Review Board (IRB) of the First Affiliated Hospital of Shandong First Medical University [YXLL-KY-2023(025)]. Formal written patient consent was acquired from the patient. Animal experiments were reviewed and approved by the Animal Ethics Committee of Shandong Center for Disease Control and Prevention (SDJK-DWLLSL-2025-018-01).

## Declaration of generative AI and AI-assisted technologies in the manuscript preparation process

During the preparation of this work, the author(s) used ChatGPT to improve the clarity and quality of the English writing. The author(s) reviewed and edited all generated text and take full responsibility for the content of the published article.

## Funding

This work was supported by the Clinical & Medical Science and Technology Innovation Program of Jinan, Shandong Province (grant number 202134040) and Cultivate Fund from The First Affiliated Hospital of Shandong First Medical University & Shandong Provincial Qianfoshan Hospital (grant number QYPY2022NSFC0802).

## Competing interests statement

The authors report there are no competing interests to declare.

## Data Availability

All data supporting the findings of this study have been deposited in Figshare and are publicly available at the following DOI: 10.6084/m9.figshare.31729438, which includes the Genbank files of pLZ002-sGFP, pSZU941, pSZU941-GFP, EU215432, pGGTOX, the raw sequencing reads of all mutated strains. pPROBE-GT was a gift from Steven Lindow (Addgene plasmid # 37814; http://n2t.net/addgene:37814; RRID:Addgene_37814).
