# Supplementary material for: Plasmid encoded mrk gene cluster promotes biofilm formation, environmental persistence, and invasiveness in Salmonella Thompson
Source: Biofilm. 2026 Aug 26;12:100394. doi: 10.1016/j.bioflm.2026.100394 (PMC13545377; doi:10.1016/j.bioflm.2026.100394)
Supplement: Supplementary file 2 [file mmc2.docx]

T**able S1. Primers used in this study.**

| **Primers** | **Sequences (5’-3’)** | **Note** |
| --- | --- | --- |
| BamH-373mrk-R | GCGCGGATCCGTCAAAGAATGCTATTTATTCGCCGACAAATATACA | Promoter of *mrk* gene cluster activity assay |
| HindIII-373mrk-F | GCGCAAGCTTGGCTTGCTGCGCTGTAAACAACCA |  |
| HindIII-950-mrk-F | GCGCAAGCTTCAGTAGCTGAACGGGAGGGACAGC |  |
| BamH-950mrk-R | GCGCGGATCCGTCAAAGAATGCTATTTATTCGCCGACAAATATACA |  |
| probe_mcs_F | GGAATTGGGGATCGGAAGCT |  |
| probe_mcs_R | TCACCTTCACCCTCTCCACT |  |
| SpeI_mrkA | AATACTAGTCAACAGTGGTATCAGCTGCAGTTTTAGAGCTAGAAATAGC | Construction of pCasCure-N20*_mrkA_* |
| mrkA_N20 | CAACAGTGGTATCAGCTGCA |  |
| XbaI-R | CTGGTTTCTAGAACTAGTGGA |  |
| N20_188R | ACGCATTGATTTGAGTCAGC |  |
| mrkA-1F | GGTAAACGTTCGCATCGCTG | amplification of *mrkA* by PCR |
| mrk-locL | GCATTCTTTGACGCCGATAGC |  |
| mrk-locR | TTAATTATAAACTAATTCCCACGTTGC | amplification of *mrkABCDF* with mrk-locL |
| IncX1-F | TCCAGGTTGCCGTAATCACC | Amplification of IncX1 replicate |
| IncX1-R | ATGGGCTGTATTCTGGCTGG |  |
| M13F | TGTAAAACGACGGCCAGT | verification of *mrkABCDF* gene cluster cloned in pEASY-T&B vector |
| M13R | CAGGAAACAGCTATGAC |  |
| glmS-TR | CAGAGAAAGCGCTCATCGAT | Verification of *sfGFP* inserted downstream of *glmS* |
| glmS-TF | TCATTGAGATGCCGCATGTG |  |
| GFP-AC-F | CAGAGTCGGCCAAGGGACCGGCAGTTTACC |  |
| GFP-AC-R | GGTAAACTGCCGGTCCCTTGGCCGACTCTG |  |
| Szu941-rev | AGGGTTATCCCGATCAACTGCAGTCTAGA | Linearization of the pSZU941‑GFP plasmid backbone |
| Szu941-for | TGATCGGGATAACCCTGAGCTCGCTTGG |  |
| APR-505F | TCGGTCAGCTTCTCAACCTT | Amplification of *aac(3’)-IV* |
| APR-505R | ACCAACTTGCCATCCTGAAG |  |
| pGG-mrk1F | GGCTACCGTCTCAGGATGGTTTGCCGCAGAAAATTTATTCA | Amplification of homologous fragments |
| pGG-mrk1R | GGCTACCGTCTCAGTGGGTTTCCGCAGTCATCAATCGA |  |
| pGG-mrk2R | GGCTACCGTCTCAATGGATATCCGGGTTATGGCCGTG |  |
| pGG-mrk2F2 | GGCTACCGTCTCAGGAGTCGTAACTAAGGGCAGGAAGTG |  |
| pGG-rm-mrk1F-P | GGCTACCGTCTCAGGATGTAAAACCGTGATGCTGGCG | Amplification of homologous fragments to eliminate *mrkCD* genes |
| pGG-rm-mrk1R-P | GGCTACCGTCTCAGTGGCAGATAGGCGCTGCTGTTAT |  |
| pGG-rm-mrk2F-P | GGCTACCGTCTCAGGAGCACTACCGCTACCATGGGC |  |
| pGG-rm-mrk2R-P | GGCTACCGTCTCAATGGCGCCGGGACAATTTTATTACTCT |  |
| luci-szu-F | AGGCACCGATAACCCTGAGCTCGCTTGG | Linearization of the pSZU941 plasmid backbone |
| luci-szu-R | GCGACCAAGCCCAAATCGATAATGCTAGCA |  |
| szu-luci-F | ATTTGGGCTTGGTCGCCTGAGAAGTTACG | Linearization of the pLuci plasmid backbone |
| szu-luci-R | AGGGTTATCGGTGCCTGACTGCGTTAG |  |
| lucif-end-F | GTGGTGTTGTGTTCGTGGAC | Verification of luciferase encoding gene cloning |
| ggTox-ResF | CATGAGCTCACTAGTGGATCCC | verification of construction of pGGTOX variants |
| ggTox-ResR | GCCTCGAGAATTCTGACGTCT |  |
| Bsa1-mrk-up | GCTCACTAACTCGCTACGCT |  |
| Bsa1-mrk-down | AAACGCGCTAACTGATCACA |  |
| gyrA-F | CTCTACTCCCAGACCCAGCT | RT-qPCR of *mrk* gene cluster induced by antibiotics |
| gyrA-R | CGCACGAACGCTGAAATGAT |  |
| qMrkC-F | CCTGCGGTTGAACATTTCGG |  |
| qMrkC-R | TTCGCCATCCAGCCAAGATT |  |
| qMrkD-F | TACACCTCCTACGACTGGGA |  |
| qMrkD-R | ATTTTTCCCGCTCAGCACTG |  |
| qMrkF-F | ACCCTGGCACTGTCAAACTG |  |
| qMrkF-R | AAAACCATCGACCCAGCGC |  |
| qMrkA-F | TACCGCGCTGACCAACAAAA |  |
| qMrkA-R | GAAACGCGCACCATCAGCTA |  |
| MrkB-R | CGCCAGCATCACGGTTTTAC |  |
| mrkB-F | AGTCTCGCCATCAACAACCC |  |
