# Supplementary material for: Plasmid encoded mrk gene cluster promotes biofilm formation, environmental persistence, and invasiveness in Salmonella Thompson
Source: Biofilm. 2026 Aug 26;12:100394. doi: 10.1016/j.bioflm.2026.100394 (PMC13545377; doi:10.1016/j.bioflm.2026.100394)
Supplement: Supplementary file 3 [file mmc3.docx]

Table S2. Fold changes, unadjusted *P* values, and Benjamini–Hochberg-adjusted q values for all 45 RT-qPCR gene–treatment comparisons.

| **drug** | **concentration (μg/mL)** | **gene** | **n** | **mean_log2fc** | **sd_log2fc** | **fold change** | ***P* value** | **q value** |
| --- | --- | --- | --- | --- | --- | --- | --- | --- |
| Ceftazidime | 0.03 | *mrkA* | 3 | 2.576666667 | 0.897819767 | 5.965597602 | 0.038168648 | 0.215600096 |
| Ceftazidime | 0.03 | *mrkB* | 3 | 1.679 | 0.848458013 | 3.202059243 | 0.075595477 | 0.233169867 |
| Ceftazidime | 0.03 | *mrkC* | 3 | 1.372666667 | 0.318123142 | 2.58948763 | 0.017436653 | 0.215600096 |
| Ceftazidime | 0.03 | *mrkD* | 3 | 0.828 | 0.377929888 | 1.775222675 | 0.062957829 | 0.233169867 |
| Ceftazidime | 0.03 | *mrkF* | 3 | 1.647666667 | 1.01612614 | 3.133264726 | 0.106840543 | 0.285475314 |
| Ceftazidime | 0.0625 | *mrkA* | 3 | 1.611 | 1.404440458 | 3.054634996 | 0.185312458 | 0.388143365 |
| Ceftazidime | 0.0625 | *mrkB* | 3 | 1.746 | 2.815452362 | 3.354272738 | 0.395156874 | 0.523001745 |
| Ceftazidime | 0.0625 | *mrkC* | 3 | 0.947 | 1.757509317 | 1.927859615 | 0.4491984 | 0.546322379 |
| Ceftazidime | 0.0625 | *mrkD* | 3 | -0.137666667 | 2.667626348 | 0.908988113 | 0.936921161 | 0.949354944 |
| Ceftazidime | 0.0625 | *mrkF* | 3 | 2.702 | 4.634832791 | 6.507033585 | 0.418916947 | 0.538607503 |
| Ceftazidime | 0.125 | *mrkA* | 3 | 1.016333333 | 1.225357227 | 2.022771467 | 0.287363968 | 0.472850998 |
| Ceftazidime | 0.125 | *mrkB* | 3 | 0.869 | 0.375902913 | 1.8263965 | 0.057083778 | 0.233169867 |
| Ceftazidime | 0.125 | *mrkC* | 3 | 0.282 | 0.712608588 | 1.215879283 | 0.563858537 | 0.646586901 |
| Ceftazidime | 0.125 | *mrkD* | 3 | -0.850666667 | 0.815257832 | 0.55452843 | 0.212457303 | 0.415677331 |
| Ceftazidime | 0.125 | *mrkF* | 3 | -1.030333333 | 1.388006604 | 0.489597015 | 0.327306402 | 0.490959603 |
| Levofloxacin | 0.06 | *mrkA* | 3 | 1.641 | 0.842318823 | 3.11881937 | 0.077723289 | 0.233169867 |
| Levofloxacin | 0.06 | *mrkB* | 3 | 0.967 | 1.270435752 | 1.954771533 | 0.318116275 | 0.490959603 |
| Levofloxacin | 0.06 | *mrkC* | 3 | -0.082333333 | 1.988504547 | 0.944528783 | 0.949354944 | 0.949354944 |
| Levofloxacin | 0.06 | *mrkD* | 3 | -1.363333333 | 0.185640333 | 0.388683203 | 0.006123726 | 0.215600096 |
| Levofloxacin | 0.06 | *mrkF* | 3 | 0.636 | 0.969042311 | 1.554014538 | 0.373490126 | 0.509304718 |
| Levofloxacin | 0.125 | *mrkA* | 3 | 1.639333333 | 0.42206911 | 3.115218449 | 0.021389507 | 0.215600096 |
| Levofloxacin | 0.125 | *mrkB* | 3 | -0.758333333 | 2.019506953 | 0.591178893 | 0.58217175 | 0.646586901 |
| Levofloxacin | 0.125 | *mrkC* | 3 | -0.840666667 | 0.29357509 | 0.558385481 | 0.038328906 | 0.215600096 |
| Levofloxacin | 0.125 | *mrkD* | 3 | -0.226 | 0.640833832 | 0.855002178 | 0.603481108 | 0.646586901 |
| Levofloxacin | 0.125 | *mrkF* | 3 | 0.617333333 | 0.540601825 | 1.534037057 | 0.186545307 | 0.388143365 |
| Levofloxacin | 0.25 | *mrkA* | 3 | -0.472 | 1.292315364 | 0.720964436 | 0.59167014 | 0.646586901 |
| Levofloxacin | 0.25 | *mrkB* | 3 | 0.324666667 | 0.702175429 | 1.252375045 | 0.507236565 | 0.60067488 |
| Levofloxacin | 0.25 | *mrkC* | 3 | 0.115 | 1.038978344 | 1.082975046 | 0.865667008 | 0.905930589 |
| Levofloxacin | 0.25 | *mrkD* | 3 | 0.434333333 | 0.773519446 | 1.351286262 | 0.433360583 | 0.541700728 |
| Levofloxacin | 0.25 | *mrkF* | 3 | 0.899 | 1.29300116 | 1.864772973 | 0.351669973 | 0.503136336 |
| Meropenem | 0.03 | *mrkA* | 3 | 2.394666667 | 1.132234222 | 5.258555916 | 0.067104101 | 0.233169867 |
| Meropenem | 0.03 | *mrkB* | 3 | 1.124666667 | 1.362127136 | 2.180511602 | 0.28895456 | 0.472850998 |
| Meropenem | 0.03 | *mrkC* | 3 | 1.568333333 | 0.972579217 | 2.965619144 | 0.10784623 | 0.285475314 |
| Meropenem | 0.03 | *mrkD* | 3 | 1.425666667 | 1.607779939 | 2.686386093 | 0.264360481 | 0.472850998 |
| Meropenem | 0.03 | *mrkF* | 3 | 2.053333333 | 1.032005006 | 4.150638637 | 0.074868629 | 0.233169867 |
| Meropenem | 0.0625 | *mrkA* | 3 | 0.626333333 | 0.915583056 | 1.54363679 | 0.357785839 | 0.503136336 |
| Meropenem | 0.0625 | *mrkB* | 3 | 1.239333333 | 1.523561726 | 2.360894106 | 0.294218399 | 0.472850998 |
| Meropenem | 0.0625 | *mrkC* | 3 | 1.29 | 0.447785663 | 2.445280555 | 0.037895863 | 0.215600096 |
| Meropenem | 0.0625 | *mrkD* | 3 | 0.860333333 | 0.716414219 | 1.815457722 | 0.173039126 | 0.388143365 |
| Meropenem | 0.0625 | *mrkF* | 3 | 0.813333333 | 0.576220733 | 1.757266904 | 0.134391518 | 0.335978794 |
| Meropenem | 0.125 | *mrkA* | 3 | -0.338 | 0.36230926 | 0.791137301 | 0.247505256 | 0.464072355 |
| Meropenem | 0.125 | *mrkB* | 3 | -0.854666667 | 0.757177214 | 0.55299308 | 0.189758978 | 0.388143365 |
| Meropenem | 0.125 | *mrkC* | 3 | -1.301 | 0.260294064 | 0.40584479 | 0.013081726 | 0.215600096 |
| Meropenem | 0.125 | *mrkD* | 3 | -2.086666667 | 0.678475743 | 0.235424004 | 0.033480667 | 0.215600096 |
| Meropenem | 0.125 | *mrkF* | 3 | -1.716333333 | 0.705731063 | 0.304321183 | 0.052000919 | 0.233169867 |
