# Supplementary material for: Plasmid encoded mrk gene cluster promotes biofilm formation, environmental persistence, and invasiveness in Salmonella Thompson
Source: Biofilm. 2026 Aug 26;12:100394. doi: 10.1016/j.bioflm.2026.100394 (PMC13545377; doi:10.1016/j.bioflm.2026.100394)
Supplement: Supplementary file 4 [file mmc4.docx]

**Supplementary files**

**Chromosomal integration of reporter cassettes**

To generate fluorescent and bioluminescent reporter strains, sfGFP or luciferase reporter cassettes were integrated into the chromosome using an established Tn7 site-specific transposition system (Supplementary Figure S1). The system uses three components: a donor strain carrying the reporter cassette flanked by Tn7 end sequences, a helper strain carrying plasmid EU215432, which encodes Tn7 transposase, and a recipient strain containing the chromosomal Tn7 attachment site downstream of *glmS*.

For construction of the sfGFP reporter plasmid, the sfGFP coding sequence was amplified from pLZ002-sGFP using the primer pairs GFP-AC-R/sfFP-BamHI and GFP-AC-F/sfFP-KpnI. This PCR design removed the KpnI restriction site. The amplified sfGFP fragment was inserted into pSZU941 by BamHI/KpnI digestion and ligation, generating pSZU941-sfGFP.

For construction of the luciferase reporter plasmid, the luciferase coding sequence was amplified from pLuci using primers szu-luci-F and szu-luci-R. Plasmid pSZU941 was linearized using primers luci-Szu-F and luci-Szu-R. The luciferase fragment and linearized pSZU941 vector were assembled using In-Fusion cloning to generate pSZU941-Luci.

The recombinant plasmids pSZU941-sfGFP and pSZU941-Luci were separately introduced into *E. coli* WM3064 to generate donor strains. Donor, recipient and helper strains were then combined by triparental conjugation. The Tn7 transposase supplied by the helper plasmid mediated insertion of the reporter cassette into the chromosomal Tn7 attachment site downstream of *glmS.*

After conjugation, the mating mixtures were plated on apramycin-containing medium without DAP. The DAP-dependent donor and helper strains were thereby counter-selected, while recipient strains containing the reporter cassette were selected. Candidate colonies were evaluated by sfGFP fluorescence or luciferase-dependent bioluminescence following the addition of luciferin. Correct chromosomal integration was verified by PCR and confirmed by Sanger sequencing.

**Figure S1. Tn7-mediated chromosomal integration of sfGFP and luciferase reporter cassettes.** The donor strain, *E. coli* WM3064, carries pSZU941-sfGFP or pSZU941-Luci, in which the reporter cassette is flanked by Tn7 end sequences. The recipient strain contains the chromosomal Tn7 attachment site downstream of *glmS*. A separate *E. coli* WM3064 helper strain carries plasmid EU215432, which encodes Tn7 transposase. Donor, recipient and helper strains are combined by triparental conjugation. Following conjugation, DAP-dependent donor and helper strains are counter-selected on apramycin-containing medium lacking DAP. Reporter-containing recipient strains are identified by sfGFP fluorescence or luciferase-dependent bioluminescence after addition of luciferin. Chromosomal insertion is confirmed by PCR and Sanger sequencing. The schematic was created using BioRender.
