# Supplementary material for: Plasmid encoded mrk gene cluster promotes biofilm formation, environmental persistence, and invasiveness in Salmonella Thompson
Source: Biofilm. 2026 Aug 26;12:100394. doi: 10.1016/j.bioflm.2026.100394 (PMC13545377; doi:10.1016/j.bioflm.2026.100394)
Supplement: Supplementary file 5 [file mmc5.docx]

**Figure S2. Antibiotic-dependent transcriptional responses of the mrk genes.**
Relative expression of mrkA, mrkB, mrkC, mrkD, and mrkF was measured by RT-qPCR after exposure to levofloxacin, ceftazidime, or meropenem at the indicated concentrations. Expression was normalized to gyrA and calibrated to the untreated LB control using the 2^-ΔΔCt method. **A**, Heatmap showing the mean log2 fold change from three independent biological replicates. Red and blue indicate increased and decreased expression, respectively. Black dots indicate gene-treatment combinations with concordant expression direction in all three replicates and a log2 fold-change standard deviation of no more than 1. B, Individual biological replicates and mean +/- SD for the reproducible gene-treatment combinations indicated in A. n = 3 independent biological experiments.
